# A simulation-driven prediction model for state of charge estimation of electric vehicle lithium battery

**DOI:** 10.1016/j.heliyon.2024.e30988

**Published:** 2024-05-09

**Authors:** Jinrui Zhang, Chenqi Song, Jiawei Xiang

**Affiliations:** a College of Mechanical and Electrical Engineering, Wenzhou University, Wenzhou, 325035, China; b Pingyang Institute of Intelligent Manufacturing, Wenzhou University, Wenzhou, 325035, China; c Wenzhou Key Laboratory of Advanced Equipment Dynamics and Intelligent Diagnosis-Maintenance, Wenzhou, 325035, China

**Keywords:** Lithium-ion battery, State of charge, Parameter identification, Simulink simulation, Unscented kalman filter

## Abstract

Accurately predicting the state of charge (SOC) of lithium-ion batteries in electric vehicles is crucial for ensuring their stable operation. However, the component values related to SOC in the circuit typically require estimation through parameter identification. This paper proposes a three-stage method for estimating the SOC of lithium batteries in electric vehicles. Firstly, the parameters of the constructed second-order RC circuit are identified using the Forgetting Factor Recursive Least Squares (FFRLS) method. Secondly, an innovative approach is employed to construct a battery simulation model using modal-data fusion method. Finally, the predicted values of the simulation model are corrected using the unscented Kalman filter (UKF). Validation through datasets demonstrates the high precision of this method in parameter identification. Moreover, in the comparison of SOC prediction corrections with Particle Filter (PF), Extended Kalman Filter (EKF), and the proposed UKF on simulated prediction data and experimental test data. The proposed method achieves the lowest root mean square error (RMSE) of 0.0025 for simulation prediction data and 0.0186 for experimental test data. It also maintained its error within 5 % on actual data.

## Highlights

• Innovative three-step method applied for precise SOC estimation in EV batteries.
• Introduction of a novel battery simulation model through modal-data fusion.
• Use of UKF for accurate correction of SOC prediction values.
• Consistent error management within 5 % on actual experimental test data.

## 1 Introduction

As the demand for an improved quality of life gradually increases, so too does the demand for vehicles. This surge contributes to the energy and environmental crises we face today [1]. The demand for vehicles has increased due to the desire for a better quality of life. However, this surge has exacerbated the energy and environmental crises we face today. Therefore, there has been an increase in the pursuit of new materials, such as nano-related [2,3] and lithium-ion new energy sources [4,5]. According to researchers, lithium-ion batteries are being considered as a potential solution for energy storage in transportation, with the aim of reducing environmental pollution caused by the energy crisis. In recent years, lithium-ion batteries with high energy density, charge-discharge performance and stability are usually used as a new choice for Electric Vehicle (EV) batteries, as well as a power source for electric vehicles, and the use of lithium-ion batteries can reduce environmental pollution and save energy [6,7], so they are favored by people.

However, it is important to note that a battery is a complex system of chemical reactions. Its structure comprises various components, including positive and negative electrodes [8], model [9], and internal medium [10]. The electrolyte's positive and negative ions move between the electrodes due to the electric current, leading to chemical reactions. However, it is important to note that batteries are chemical reaction systems that are subject to nonlinear and time-varying reactions, which can be influenced by various factors, including temperature. To ensure optimal battery performance, electric vehicles are equipped with their own battery management systems (BMS). The BMS is responsible for a range of functions, including predicting the state of charge (SOC), state of health (SOH), and remaining useful life (RUL) of the battery.

In the realm of lithium battery-related index prediction, methods for estimating State of Health (SOH) have demonstrated notable advancements. For instance, Ref. [11] introduced the ANA-LSTM neural network method, which exhibits remarkable accuracy in predicting the remaining useful life of lithium batteries, even amidst multiple influencing factors. Additionally, Ref. [12] presented a novel framework that integrates Mixers and Bidirectional Temporal Convolutional Neural Network (BTCN) for SOH estimation. Moreover, Ref. [13] proposes an enhanced modeling approach, the robust multi-time scale singular filtering-Gaussian process regression-long short-term memory (SF-GPR-LSTM) method, tailored for precise remaining capacity estimation in low-temperature environments encountered by lithium batteries.

Enlightened by the myriad prediction methods for State of Health (SOH), researchers have extended their application to predict State of Charge (SOC), an area with relatively fewer established methods. Currently, two primary SOC prediction methodologies exist. The first is model-based, involving the construction of circuit models based on battery charging and discharging characteristics, as explored by Ren et al. [14], who proposed a SOC prediction method combining unscented Kalman filtering with initial SOC acceleration convergence. While model-based approaches offer simplicity and reliability, establishing an accurate battery model is crucial for achieving highly precise SOC prediction results. On the other hand, data-driven methods predominantly utilize current and voltage data obtained from battery tests, as demonstrated by Liu et al. [15], who proposed a data-driven SOC prediction method for lithium-ion batteries based on Extended Kalman Filtering (EKF), leveraging machine learning to enhance prediction accuracy. Wu et al. [16], introduced a battery SOC prediction method based on electric vehicle trip data, integrating random forest dimensionality reduction with long short-term memory (LSTM) to enhance prediction robustness. Although widely used, the equivalent circuit model's simplicity belies the significant impact of its parameters on SOC prediction accuracy. Hence, it is imperative to consider these influences of parameters when predicting battery SOC, as emphasized by Tan et al. [17], who proposed an extended Kalman filtering recursive least squares method based on the second-order RC equivalent circuit model to enhance identification accuracy. Additionally, Hu et al. [18], proposed collaborative particle swarm optimization (MCPSO) with hybrid swarm encapsulation to identify and amplify battery parameters for collaborative updating, offering faster convergence to optimal solutions with higher accuracy. Shi et al. [19] employed the forgetting factor recursive least squares method to adaptively adjust battery parameters, enhancing identification accuracy. However, many existing methods overlook the influence on battery performance of internal parameters. Hence, it is proposed that an approach integrates the predictive power of model-based methodologies with real-time data linkage to revolutionize SOC prediction. This innovative fusion allows for rapid battery simulation and comprehensive characterization of parameter SOC relationships, culminating in highly accurate predictions.

In this paper, based on the current and voltage data tested by the lithium battery in the Urban Dynamometer Driving Schedule (UDDS), a second-order RC equivalent circuit was established to describe the dynamic operating characteristics of the battery. The battery parameters were identified by Forgetting Factor Recursive Least Square (FFRLS), and the functional relationship between SOC and each parameter was fitted. Based on the fitting function relation, a simulation model corresponding to the structure function of second-order RC equivalent circuit was established in Simulink to verify the accuracy of parameter identification. Based on the simulation results, the Unscented Kalman Filter (UKF) was used to correct the SOC prediction results, and the optimal prediction curve of battery SOC was obtained. The highlight of the present work is that we present a three-stage method for state of charge estimation of electric vehicle lithium batteries. Detail contributions are summarized as follows.

1) Development of a Refined Three-Step Methodology for SOC Estimation: An elegantly crafted three-step process was proposed that transcends traditional SOC estimation methods by integrating an advanced model-data fusion approach based on the electrical circuit simulation model. This approach ensures a more accurate, reliable, and comprehensive analysis of battery performance.
2) Advanced Parameter Identification via the FFRLS Method: A cornerstone of our research is the utilization of the Forgetting Factor Recursive Least Square (FFRLS) method for the identification of parameters in a second-order RC circuit model. This technique allows for the precise determination of the interrelationships between battery components and SOC. By meticulously identifying these parameters, bridge the gap between theoretical models and real-world data.
3) Advanced Simulation Model Based on Second-Order RC Circuit for SOC Prediction: Our work introduces a sophisticated simulation model grounded in the parameters of a second-order RC circuit model, enabling precise SOC predictions through model-data fusion. This approach effectively merges theoretical insights with empirical data, enhancing the accuracy of SOC estimates.
4) Prediction Accuracy Enhanced with Unscented Kalman Filter: The refinement of SOC predictions is achieved through the utilization of the Unscented Kalman Filter (UKF). This method corrects estimates based on real-time data, significantly improving the accuracy of our SOC predictions by accounting for the nonlinear behavior of lithium batteries.

The subsequent sections of this manuscript are as follow: Section II introduces the theoretical underpinnings and critical methodologies employed, setting the stage for a deeper exploration of our work. Section III is devoted to elucidating the proposed method, detailing the innovative approach. In Section IV, we subject our method to rigorous experimental validation, presenting the results that demonstrate its efficacy. The manuscript concludes with Section V, where we encapsulate our findings, reflect on the implications of our work, and suggest avenues for future research.

## 2 Methodology

### 2.1 Second-order RC equivalent circuit model

**Fig. 1:**
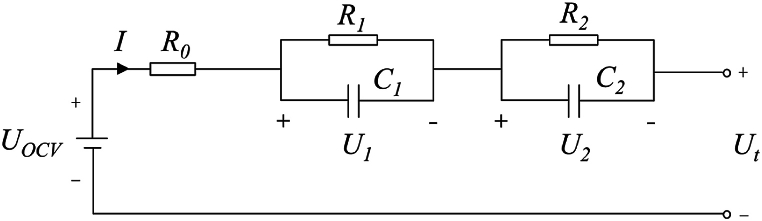
Second-order RC equivalent circuit model.

This paper employs lithium iron phosphate batteries. Based on their charge-discharge characteristics, a second-order RC equivalent circuit model is established to describe the dynamic operating behavior of the battery [20], as illustrated in Fig. 1.

The second order RC cell model mainly consists of the following parts. *I* is the battery input current, Ut is the battery output voltage.

UOCV is the open circuit voltage of the battery and has a certain correlation with SOC.

R0 is the ohm internal resistance of the battery, which blocks the circulation of current. When the battery is discharged, the ohm internal resistance is the main factor that reduces the capacity of the battery.

R1 and C1 are concentration polarization resistance and concentration polarization capacitance, respectively, which are mainly caused by the resistance of ions in the electrolyte or electrolyte to react on the electrode.

R2 and C2 are electrochemical polarization resistance and electrochemical polarization capacitance, respectively, which come from the effect of slower electrochemical reaction on the electrode.

### 2.2 Parameter identification method

The internal parameters of the battery can't be directly measured by instruments, so it is necessary to use mathematical methods to calculate, using measurable current and voltage data combined with identification algorithm to identify the internal parameters [[21], [22], [23]].

In this paper, the least square method with less computation is used to identify battery parameters, but the accuracy of the traditional least square method is low. Therefore, the recursive least square method with amnesia factor was used in this paper to identify battery parameters. The amnesia factor can effectively reduce the influence of invalid data on the identification accuracy, so as to improve the parameter identification accuracy. The parameter identification values corresponding to SOC data points were rounded, and the relationship curve between each parameter and SOC was obtained by using the fitting tool, which was applied to Simulink simulation and SOC prediction.

The FFRLS (Forgetting Factor Recursive Least Squares), as an enhancement over the conventional RLS algorithm [24]. This improvement is necessitated by the observation that the RLS method is prone to severe filtering saturation with an increase in the number of sampling instances. Such saturation leads to the parameter of algorithm estimates failing to track time-varying parameters in real-time, consequently diminishing its data correction capabilities. To address this, the FFRLS method incorporates a forgetting factor (0.95<μ < 1) into the foundation of the RLS identification algorithm. This addition aims to mitigate the accumulation of outdated data during iterative computations, thereby amplifying the feedback effect of new data. The fundamental computational formula is represented as follows in Eq. (1):

(1) $$y\left(k+1\right)=\varphi^{T}\left(k\right)\hat{\theta }\left(k\right)+e\left(k\right)$$

In this equation, y(k+1) denotes the actual system observation value; $\varphi^{T}\left(k\right)$ represents the system data variable; $\hat{\theta }\left(k\right)$ signifies the optimum estimation of the parameter variable at the instant k; and e(k) denotes the zero-mean white noise, also referred to as the innovation vector, indicating the discrepancy between optimum prediction of the current moment and the output value of the next instance.

Calculate process of FFRLS is shown at Eq. (2), *μ* is the forgetting factor, usually ranging from 0.95 to 0.99. $K_{k}$ is the gain matrix; $P_{k}$ is the error covariance matrix. Input the open-source current data and voltage data into the algorithm, you can calculate the parameter change value at each moment.

(2) $$\left\{ \begin{array}{c} K_{k}=P_{k-1}\varphi_{k}^{T}{\left[\varphi_{k}P_{k-1}\varphi_{k}^{T}+\mu \right]}^{-1}\\ P_{k}=\frac{1}{\mu }\left[I-K_{k}\varphi_{k}\right]P_{k-1}\\ {\hat{\theta }}_{k}={\hat{\theta }}_{k-1}+K_{k}\left[y_{k}-\varphi_{k}{\hat{\theta }}_{k-1}\right] \end{array}\right.$$

### 2.3 SOC prediction optimization algorithm

Unscented Kalman Filter (UKF) and Extended Kalman Filter (EKF) are two applications based on Kalman Filter principle [25,26]. Different from EKF, UKF has better accuracy and convergence. By obtaining corresponding Sigma points through Untraced Transformation (UT), UKF approximates the posterior probability density of the state [27], thus ensuring the accuracy of filtering prediction and avoiding the complexity of state transition matrix and observation matrix operation [28,29]. Here is UKF calculate process.

Step 1: Determine the initial battery system status. Initialize the mean value of the state variable ${\bar{x}}_{0}$, the initial value of the covariance matrix $P_{0}$, and the initial values of the mean and variance weights shown in Eq. (3).

(3) $$\left\{ \begin{array}{c} {\bar{x}}_{0}=E\left(x_{0}\right)\\ P_{0}=E{\left[\left(x_{0}-{\bar{x}}_{0}\right)\left(x_{0}-{\bar{x}}_{0}\right)\right]}^{T} \end{array}\right.$$

In Eq. (3), E(·) represents the mean value of each variable as shown in Eq. (4).

(4) $$\left\{ \begin{array}{c} \lambda =\alpha^{2}\left(L+\kappa \right)-L\\ W_{m}^{0}=\frac{\lambda }{L+\lambda }\\ W_{c}^{0}=\frac{\lambda }{L+\lambda }+\beta -\alpha^{2}+1\\ W_{m}^{k}=W_{c}^{k}=\frac{1}{2\left(L+\lambda \right)}，k=1:2L+1 \end{array}\right.$$

in which, $W_{m}$ and $W_{c}$ are the mean and variance weights, respectively. λ is the proportional coefficient to reduce the prediction error of system. L is the dimensionality of the state variable depends on the dimension of the state matrix. κ is the scale factor. β is a non-negative weight coefficient. α is the distribution state of control sampling points, which ranges from 10^−4^ to 1.

Step 2: Calculate the Sigma sampling sites of the state variables at time k and store them are shown in Eq. (5). ${\hat{x}}_{k}$ is the optimal estimated value of the state variable at time k; $P_{k}$ is the covariance of the state variable at time k, $x_{sigma,k}^{i}$ is the Sigma sampling point at time k.

(5) $$\left\{ \begin{array}{c} x_{\text{sigma},k}^{0}={\hat{x}}_{k}\\ x_{\text{sigma},k}^{i}={\hat{x}}_{k}+\sqrt{P_{k}\left(L+\lambda \right)}，i=1:L\\ x_{\text{sigma},k}^{i}={\hat{x}}_{k}-\sqrt{P_{k}\left(L+\lambda \right)}，i=L+1:2L \end{array}\right.$$

Step 3: Update the status time. According to Eq. (6) to Eq. (8), 2L+1 Sigma sampling points calculated in Step 2 were used to update the mean value of the state variable $x_{\text{pred},k|k-1}^{i}$ and the covariance ${\tilde{P}}_{\text{pred}}$ at time k.

(6) $$x_{\text{sigmapre},k|k-1}^{i}=f\left(x_{\text{sigma},k-1}^{i},u_{k}\right)，i=0∼2L$$

(7) $$x_{\text{pred},k|k-1}^{i}=\sum_{i=0}^{2L}W_{m}^{i}x_{\text{sigmapre},k|k-1}^{i}$$

(8) $${\tilde{P}}_{\text{pred}}=\sum_{i=0}^{2L}W_{c}^{i}\left(x_{\text{sigmapre},k|k-1}^{i}-x_{\text{pred},k|k-1}^{i}\right){\left(x_{\text{sigmapre},k|k-1}^{i}-x_{\text{pred},k|k-1}^{i}\right)}^{T}+Q_{k-1}$$

where $Q_{k-1}$ is the mean value of process noise of battery system. k|k−1 indicates that the variable is based on the time value of the variable before updating the values of the variables of the next moment. $x_{sigmapre.k|k-1}^{i}$ is the predicted value of Sigma point set based on k-1 time.

Step 4: Update the observation time and calculate the mean of observation prediction ${\hat{\bar{y}}}_{k|k-1}$ and the covariance are shown in Eq. (9) to Eq. (12).

(9) $${\hat{y}}_{k|k-1}=g\left(x_{\text{sigmapre},k|k-1}^{i},u_{k}\right)$$

(10) $${\hat{\bar{y}}}_{k|k-1}=\sum_{i=0}^{2L}W_{m}^{i}g\left(x_{\text{sigmapre},k}^{i},u_{k}\right)+R_{k-1}$$

(11) $${\tilde{P}}_{y,k}=\sum_{i=0}^{2L}W_{c}^{i}\left({\hat{y}}_{k|k-1}-{\hat{\bar{y}}}_{k|k-1}\right){\left({\hat{y}}_{k|k-1}-{\hat{\bar{y}}}_{k|k-1}\right)}^{T}$$

(12) $${\tilde{P}}_{xy,k}=\sum_{i=0}^{2L}W_{c}^{i}\left(x_{\text{sigmapre},k|k-1}^{i}-x_{\text{pred},k|k-1}^{i}\right){\left({\hat{y}}_{k|k-1}-{\hat{\bar{y}}}_{k|k-1}\right)}^{T}$$

where $R_{k-1}$ is the mean value of observation noise of battery system. ${\hat{y}}_{k|k-1}$ is the observed predicted value based on the k-1 time, ${\tilde{P}}_{y,k}$ and ${\tilde{P}}_{xy,k}$ are the observed covariances at time k.

Step 5: Calculate the unscented Kalman filter gain matrix k at time $K_{k}$ as shown in Eq. (13).

(13) $$K_{k}={\tilde{P}}_{y,k}{\tilde{P}}_{xy,k}^{-1}$$

Step 6: State covariance prediction update. Calculate the updated optimal predictive value of the state variable ${\hat{x}}_{k}$ and the optimal covariance matrix ${\hat{P}}_{k}$ at time k, as shown in Eq. (14) and Eq. (15).

(14) $${\hat{x}}_{k}=x_{\text{pred},k|k-1}^{i}+K_{k}\left(y_{k}-{\hat{\bar{y}}}_{k|k-1}\right)$$

(15) $${\hat{P}}_{k}=P_{\text{pred}}-K_{k}{\tilde{P}}_{y,k}K_{k}^{T}$$

The above six steps can complete Sigma point sampling and state prediction update in the UKF algorithm, that is, the optimal predicted value of the UKF of the battery SOC at time k can be obtained from the optimal predicted value of the state variable ${\hat{x}}_{k}$.

## 3 Proposed three-step method

**Fig. 2:**
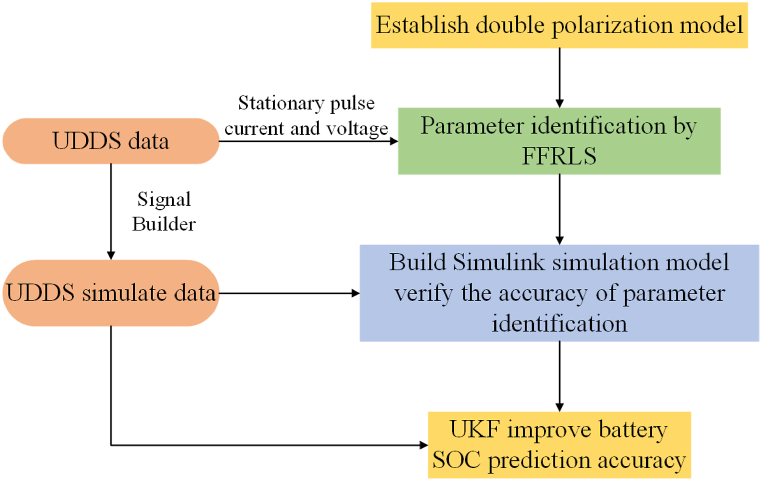
Research flow chart.

In order to better solve the SOC prediction problem of lithium-ion batteries, this paper proposes a three-step method for estimating the state of charge of lithium-ion batteries in electric vehicles. The flow chart is shown in Fig. 2, and the detailed steps are summarized as follows.

Step1: Construction of Equivalent Circuit Model and Preliminary Parameter Identification
  (a) Data Collection: Collection of foundational data from battery charging and discharging experiments, including open-circuit voltage, current, along with charging and discharging voltages and currents.
  (b) Establishment of Equivalent Circuit Model: Utilization of a second-order RC circuit model based on the collected data to express dynamic working characteristics of the battery. This model incorporates elements such as open-circuit voltage and current to reflect the charging and discharging characteristics.
  (c) Preliminary Parameter Identification: Application of the Forgetting Factor Recursive Least Squares method for initial identification of parameters within the second-order RC circuit model.
Step 2: Precise Parameter Identification and Simulation Verification
  (a) Precise Parameter Identification: Continuation of the FFRLS method application for precise parameter identification within the circuit model, ensuring model accuracy and practicality.
  (b) Simulink Simulation: Engagement with Simulink tool of MATLAB for simulation of the established second-order RC circuit model as a dynamic system. Verification of equivalent circuit model and parameter identification accuracy and feasibility through simulation ensures the model accurately reflects dynamic working characteristics for batteries.
Step 3: State Estimation and Optimization
  (a) SOC Prediction: Design of an SOC (State of Charge) prediction algorithm leveraging the Unscented Kalman Filter. Utilization of UKF for battery state estimation, focusing on SOC prediction.
  (b) Simulation and Optimization: Integration of Simulink simulation with UKF for further optimization and correction of simulation results, enhancing SOC prediction accuracy.

Through these steps, not only is the construction and validation of an accurate second-order RC circuit model that reflects dynamic working characteristics of batteries achieved, but also the effective prediction of the SOC is facilitated, providing accurate methodological support for battery management systems.

## 4 Validation for proposed method

### 4.1 Introduction of dataset

Urban Dynamometer Driving Schedule (UDDS), referred to as FTP72 condition, is a test procedure used by the US environmental protection agency in 1972 to certify the vehicle plaque, and later it is often used as one of the test conditions for lithium batteries [[30], [31], [32]]. In this paper, the open-source data of battery current and voltage measured in UDDS of the United States was adopted on the scientific research data management and sharing platform of Elsevier company Mendeley data.

**Fig. 3:**
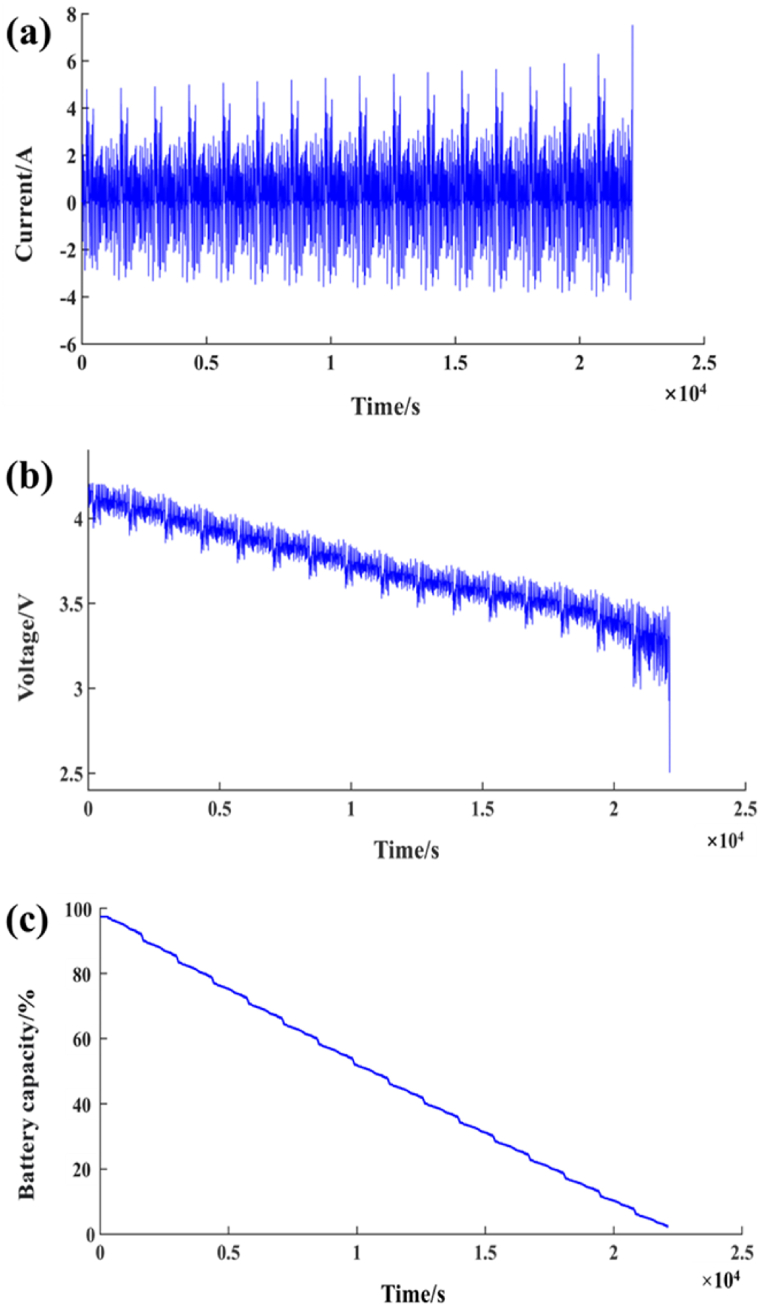
Battery test data under UDDS conditions: (a) Current; (b) Voltage; (c) Battery capacity.

The data source of this paper is the UDDS in the United States on research data management and sharing platform of Mendeley. The current, voltage, battery capacity and other open-source data of Panasonic 18650PF lithium battery (battery type is lithium iron phosphate battery) measured in UDDS are used as research data, as shown in Fig. 3(a) to Fig. 3(c).

The data link is:

https://data.mendeley.com/datasets/wykht8y7tg/1.

The parameters of Panasonic 18650PF lithium battery is shown in Table 1.

**Table 1:** Battery parameters value.

Parameters	Value
Rated current/A	3
Rated voltage/V	3.75
Peak voltage/V	4.2
Cut off voltage/V	2.5
Rated capacity/Ah	2.9
Actual capacity/Ah	2.7728

### 4.2 Parameter identification based on FFRLS

**Fig. 4:**
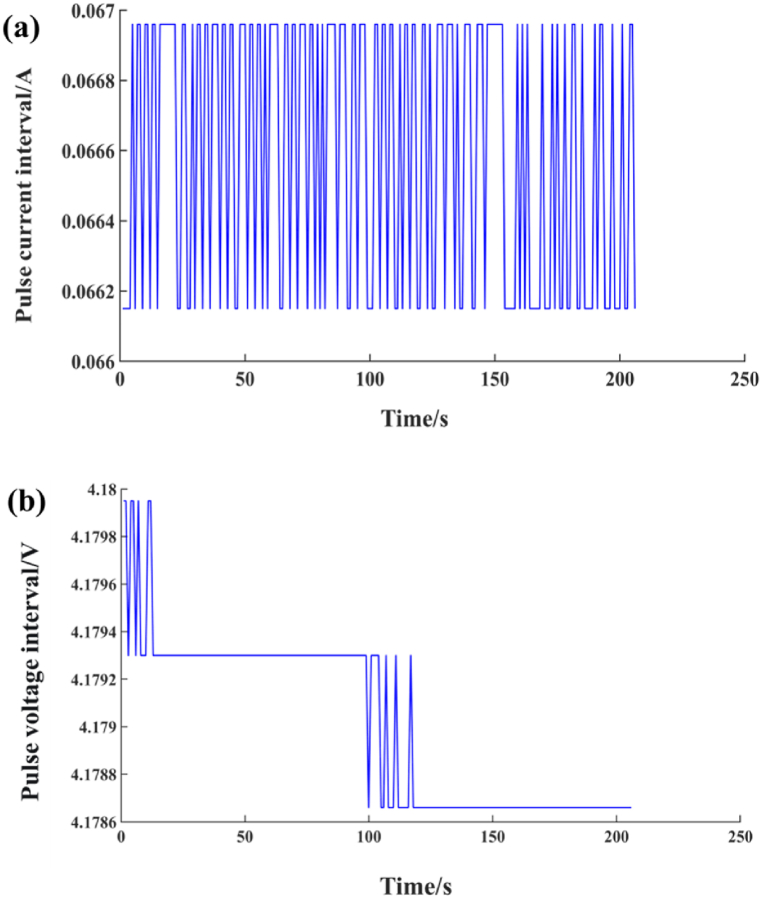
UDDS current and voltage range. (a) Current range; (b) Voltage range.

The data derived from the UDDS operational conditions exhibit notable regularity. Notably, a 10 % reduction in battery capacity corresponds to a consistent pulse current value, as illustrated in Fig. 4(a), and falls within a defined voltage range, as shown in Fig. 4(b). The variance between consecutive pulse current measurements remains remarkably stable, ranging between 0.00081 and 0.00083. Drawing on these findings, this study employs the specified current and voltage parameters in conjunction with the FFRLS algorithm to facilitate the online identification of battery parameters.

According to voltage law and current law of Kirchhoff, the battery state equation based on the second-order RC equivalent circuit model can be obtained, as shown in Eq. (16).

(16) $$\left\{ \begin{array}{c} {\dot{U}}_{1}=\frac{I}{C_{1}}-\frac{U_{1}}{R_{1}C_{1}}\\ U_{t}=U_{\text{OCV}}-U_{1}-U_{2}-IR_{0}\\ {\dot{U}}_{2}=\frac{I}{C_{2}}-\frac{U_{2}}{R_{2}C_{2}} \end{array}\right.$$

The transfer function of the battery system can be obtained by applying Laplace transform to Eq. (16), as shown in Eq. (17).

(17) $$G\left(s\right)=‐\frac{R_{0}s^{2}+\frac{1}{R_{S}C_{S}}\left(R_{0}R_{S}C_{S}+R_{0}R_{P}C_{P}+R_{P}R_{S}C_{S}+R_{S}R_{P}C_{P}\right)s+\frac{R_{0}+R_{S}+R_{P}}{R_{S}C_{S}R_{P}C_{P}}}{s^{2}+\frac{R_{S}C_{S}+R_{P}C_{P}}{R_{S}C_{S}R_{P}C_{P}}s+\frac{1}{R_{S}C_{S}R_{P}C_{P}}}$$

Make $s=\frac{2}{Vt}\cdot \frac{1-z^{-1}}{1+z^{-1}}$, the transfer function is changed bilinear. Make $\tau_{S}=R_{S}C_{S}$, $\tau_{P}=R_{P}C_{P}$, a linearized equation of state can be obtained, as shown in Eq. (18) and Eq. (19).

(18) $$G\left(z^{-1}\right)=\frac{a_{3}+a_{4}z^{-1}+a_{5}z^{-2}}{1-a_{1}z^{-1}-a_{2}z^{-1}}$$

(19) $$\left\{ \begin{array}{c} a_{1}=-\frac{2△t-8\tau_{S}\tau_{P}}{△t^{2}+2△t\left(\tau_{S}+\tau_{P}\right)+4\tau_{S}\tau_{P}}\\ a_{2}=-\frac{△t^{2}-2△t\left(\tau_{S}+\tau_{P}\right)+4\tau_{S}\tau_{P}}{△t^{2}+2△t\left(\tau_{S}+\tau_{P}\right)+4\tau_{S}\tau_{P}}\\ a_{3}=-\frac{△t^{2}\left(R_{0}+R_{S}+R_{P}\right)+2△t\left(R_{0}\tau_{S}+R_{0}\tau_{P}+R_{S}\tau_{P}+R_{P}\tau_{S}\right)+4R_{0}\tau_{S}\tau_{P}}{△t^{2}+2△t\left(\tau_{S}+\tau_{P}\right)+4\tau_{S}\tau_{P}}\\ a_{4}=-\frac{2△t^{2}\left(R_{0}+R_{S}+R_{P}\right)-8R_{0}\tau_{S}\tau_{P}}{△t^{2}+2△t\left(\tau_{S}+\tau_{P}\right)+4\tau_{S}\tau_{P}}\\ a_{5}=-\frac{△t^{2}\left(R_{0}+R_{S}+R_{P}\right)-2△t\left(R_{0}\tau_{S}+R_{0}\tau_{P}+R_{S}\tau_{P}+R_{P}\tau_{S}\right)+4R_{0}\tau_{S}\tau_{P}}{△t^{2}+2△t\left(\tau_{S}+\tau_{P}\right)+4\tau_{S}\tau_{P}} \end{array}\right.$$

The linear equation of state is discretized, as shown in Eq. (20).

(20) $$U_{t,k}=\left(1-a_{1}-a_{2}\right)U_{\text{OCV},k}+a_{1}U_{t,k-1}+a_{2}U_{t,k-2}+a_{3}i_{k}+a_{4}i_{k-1}+a_{5}i_{k-1}$$

Then the data matrix $\varphi_{k}$ and parameter matrix $\theta_{k}$ of the battery system can be obtained, and the input-output equation of FFRLS can be established, as shown in Eq. (21) and Eq. (22).

(21) $$\varphi_{k}=\left[\begin{array}{cccccc} 1 & U_{t,k-1} & U_{t,k-1} & i_{k} & i_{k-1} & i_{k-2} \end{array}\right]$$

(22) $$\theta_{k}=\left[\begin{array}{cccccc} \left(1-a_{1}-a_{2}\right)U_{\text{OCV},k} & a_{1} & a_{2} & a_{3} & a_{4} & a_{5} \end{array}\right]$$

Therefore, the input-output equation of FFRLS is shown in Eq. (23), and the calculation equation is shown in Eq. (2).

(23) $$y_{k}=\varphi_{k}\cdot \theta_{k}$$

Above all, FFRLS was employed to identify the parameters. This method allows for obtaining the variation values of battery parameters throughout all times, along with changes in current and voltage. However, the resulting data volume post-identification is considerable. To mitigate the computational complexity associated with Simulink simulation and UKF prediction of SOC, it becomes imperative to establish the relationship between parameters and SOC changes. In this study, parameter identification values were selected with SOC ranging from 0 to 100 % in increments of 10 %. Post FFRLS parameter identification, the mathematical relationship between each parameter and SOC is delineated in Eq. (24) to Eq. (29). The parameter identification values for each parameter are presented in Table 2.

**Table 2:** Battery parameter identification values.

SOC/%	OCV/V	*R*_0_/mΩ	*R*_1_/mΩ	*C*_1_/F	*R*_2_/mΩ	*C*_2_/F
0	3.308	11.35	45.79	20.58	25.35	794.38
10	3.402	9.53	40.31	18.59	9.29	929.89
20	3.522	8.90	34.76	17.28	4.55	1811.97
30	3.592	8.76	31.93	18.05	4.04	2078.22
40	3.651	7.04	32.95	21.52	3.79	2208.21
50	3.731	7	32.46	20.39	4.67	2420.39
60	3.838	9.73	35.08	20.37	6.59	2407.27
70	3.906	9.60	35.64	20.10	7.07	2520.16
80	3.994	8.60	34.43	19.25	7.30	2461.68
90	4.079	7.57	34.09	18.54	6.83	1971.30
100	4.127	9.33	37.14	20.01	8.94	1342.95

An interesting phenomenon can be seen from Table 2. After the battery discharge, the resistance values of internal polarization resistance R1 and R2 both showed a sudden drop, and then tended to be stable, without sudden fluctuations. The capacitance value of C1 increases first after the battery discharge. When the SOC reaches 50 %, the capacitance value of C1 decreases significantly. When the SOC drops to 10 %, the capacitance value of C1 rises again to the state before discharge. The polarization capacitance C2 value increases to a stable value after the battery is discharged, and continues to decline when the SOC reaches 50 %. Thus, when SOC = 50 %, most of the parameters are mutated. That is to say, when the battery capacity reaches 50 %, the chemical reaction rate and effect inside the battery will be affected to some extent.

(24) $$U_{\text{OCV}}=9.975SOC^{6}-31.1SOC^{5}+35.03SOC^{4}-16.83SOC^{3}+2.855SOC^{2}+0.8911SOC+3.306$$

(25) $$R_{0}=-0.00192SOC^{6}-0.00129SOC^{5}+0.00633SOC^{4}+0.00443SOC^{3}-0.00568SOC^{2}-0.00274SOC-0.00763$$

(26) $$R_{1}=0.0005631SOC^{7}-0.000003241SOC^{6}+0.000004951SOC^{5}+0.001357SOC^{4}-0.006449SOC^{3}+0.0006511SOC^{2}+0.004926SOC+0.03337$$

(27) $$C_{1}=-0.6405SOC^{6}+0.01415SOC^{5}+4.077SOC^{4}-0.687SOC^{3}-6.273SOC^{2}+1.243SOC+21.02$$

(28) $$R_{2}=-0.0004941SOC^{7}+0.001879SOC^{6}+0.001041SOC^{5}-0.003049SOC^{4}-0.0041SOC^{3}+0.002626SOC^{2}+0.004296SOC+0.004869$$

(29) $$C_{2}=‐172.9SOC^{5}‐64.84SOC^{4}+377SOC^{3}‐469.8SOC^{2}+222.6\text{SOC}+2427$$

### 4.3 Simulink Simulation based on model

**Fig. 5:**
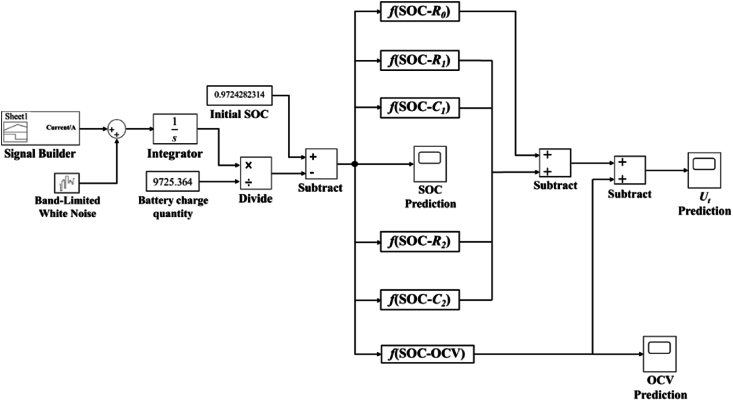
Battery simulation model framework.

The operational condition of the battery is primarily indicated by changes in the terminal voltage [33,34]. Therefore, Simulate the working state of the battery using a battery system simulation model, and verify the accuracy of parameter identification and the robustness of the second-order RC equivalent circuit model. According to the existing mathematical relationship between each parameter and SOC, the full response law of resistance-capacitance network, and the charge-discharge characteristics of the battery. Simulink is employed here to simulate battery charging and discharging characteristics. Moreover, Simulink is simple to perform without consider the chemical reactions in batteries. Therefore, a simulation model based on second-order RC equivalent circuit is established in Simulink. Fig. 5 shows the overall architecture of the model. The SOC simulation module in the model is composed according to the definition of SOC, as shown in Eq. (30).

(30) $$SOC_{k}=SOC_{0}-\frac{\eta \int Idt}{Q}$$

in Eq. (30), $SOC_{0}$ is the initial battery SOC, ***η*** is the charging and discharging efficiency. For lithium batteries, the charging and discharging efficiency is basically 1, and ***Q*** is the battery power.

The input signal of the simulation prediction model is the UDDS test current, and the Gaussian band-limited white noise signal module is added to simulate the real UDDS test condition. Because the normally distributed random numbers generated by the Gaussian band-limited white noise module are applicable to continuous or mixed systems [35]. The battery system is a continuous nonlinear system, so adding noise can express the actual working state of the battery more clearly.

**Fig. 6:**
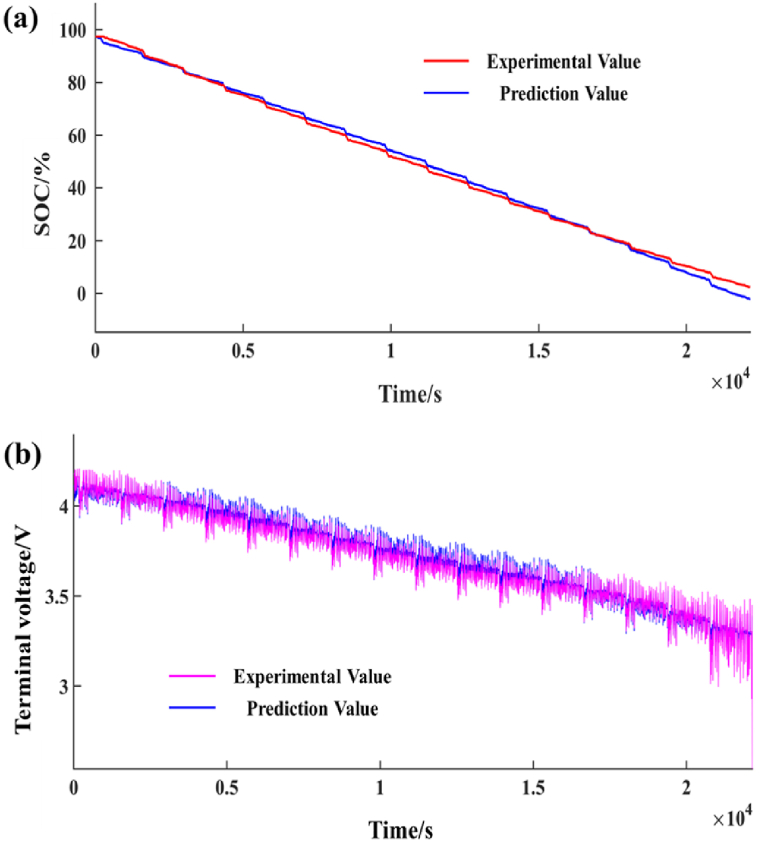
Simulation and experiment results. (a) SOC results; (b)Terminal voltage value results.

**Fig. 7:**
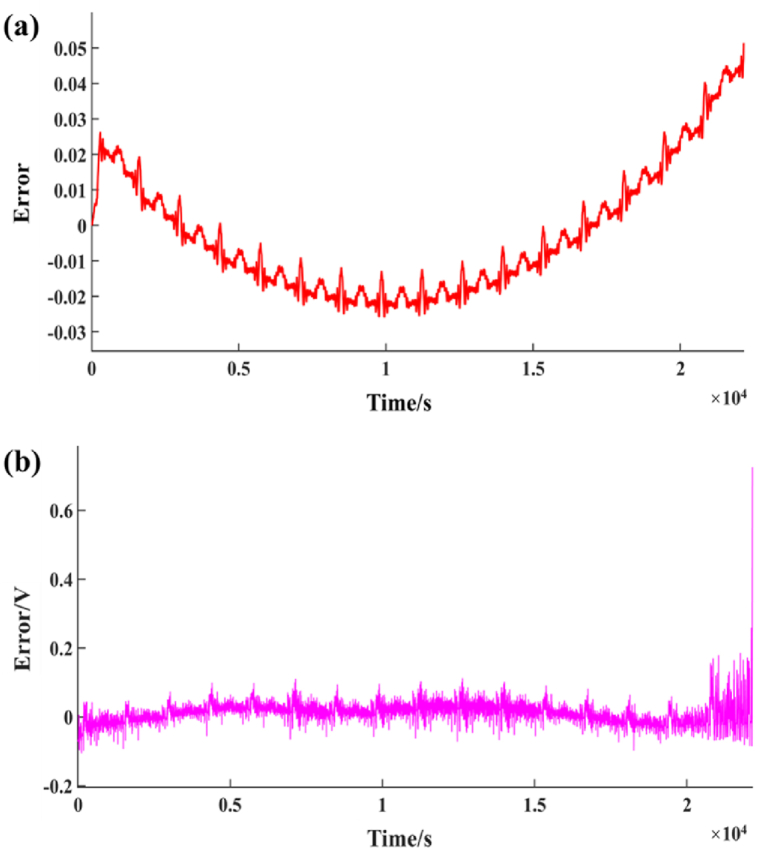
Error between simulation and experiment results. (a) SOC error; (b)Terminal voltage error.

The actual capacity of the battery used in this paper is 2.7015Ah, so the total charge and discharge charge of the battery is 9725.364C. The initial capacity of the battery before the test, that is, the initial SOC, is 97.24 %. These are used as the initial amount of the battery system to simulate the changes in terminal voltage and SOC of the battery system. Fig. 6(a) and (b) shows the comparison between simulation and experimental results, Fig. 7(a) and (b) shows the error between them.

As can be seen from Fig. 7(a) and (b), there are still some errors in simulating the working state of battery SOC and simulated battery by using Simulink. The initial simulation results are close to the experimental results, but when the battery capacity is gradually exhausted, the simulation errors of SOC gradually increase, even up to 0.054. The simulation error of terminal voltage fluctuates between 0 and 0.5V, but when the battery capacity is about to be exhausted, the error can reach up to 0.8V.

It can be found from the experimental test results that when the battery SOC drops to about 20 %, the battery SOC decreases gradually and slowly, and the frequency of decline gradually decreases. The average amplitude of the simulation SOC is basically similar, but when the battery capacity is close to 20 %, there is a big difference between the predicted results of the simulation and the experimental results. Generally, when measuring SOC, the voltage of the battery is not affected. However, if the SOC is less than 20 %, the characteristics of the battery will be sharply changed by the weakness of electrolyte consumption and side reactions in electrolytic reaction.

Therefore, when the battery capacity is low, the simulation model will have a large error in predicting SOC and battery working state. When the SOC prediction error is large, it will also have a great impact on the charging and discharging state of the battery.

### 4.4 UKF improve accuracy of SOC prediction

From the analysis of the previous results, it is necessary to consider using UKF to correct the predicted values of the model. According UKF to predict SOC, the lithium battery system should be discretized to obtain the state equation and observation equation of the discretized system, which present in Eq. (31).

(31) $$\left\{ \begin{array}{c} x_{k}=f\left(x_{k-1},u_{k}\right)=A_{k}x_{k-1}+{Β}_{k}u_{k}+\omega_{k}\\ y_{k}=g\left(x_{k},u_{k}\right)=C_{k}x_{k}+D_{k}u_{k}+\upsilon_{k} \end{array}\right.$$

in which, $x_{k}$ is the state equation representing the state of the battery system at time k, $y_{k}$ is the observation equation representing the observed output of the battery system at time k, such as the battery terminal voltage. $u_{k}$ represents the input to the battery system, such as the battery current, $w_{k}$ and $v_{k}$ are independent process noise and observation noise, respectively. $A_{k}$ and ${Β}_{k}$ are the state matrices of the battery system, $C_{k}$ and $D_{k}$ are the observation matrices of the battery system.

According to Eq. (30), the state space model of the battery system can be expressed in the form of discrete time equation, as shown in Eq. (32).

In Eq. (32), $U_{i.k}$ is the terminal voltage at time k of two RC networks. $I_{k}$ is the current at moment k of the circuit.

(32) $$\left\{ \begin{array}{c} x_{k}=f\left(x_{k-1},u_{k}\right)=\left[\begin{array}{ccc} 1 & 0 & 0\\ 0 & \exp\left(-\frac{T}{R_{1}C_{1}}\right) & 0\\ 0 & 0 & \exp\left(-\frac{T}{R_{2}C_{2}}\right) \end{array}\right]\times \left[\begin{array}{c} SOC_{k-1}\\ U_{1,k-1}\\ U_{2,k-1} \end{array}\right]+\left[\begin{array}{c} -\frac{\eta T}{Q}\\ R_{1}[1-\exp\left(-\frac{T}{R_{1}C_{1}}\right)]\\ R_{2}[1-\exp\left(-\frac{T}{R_{2}C_{2}}\right)] \end{array}\right]\times I_{k}+\omega_{k}\\ y_{k}=g\left(x_{k},u_{k}\right)=U_{OCV,k}\left(SOC_{k}\right)-U_{1}-U_{2}-I_{k}R_{0}+\upsilon_{k} \end{array}\right.$$

**Fig. 8:**
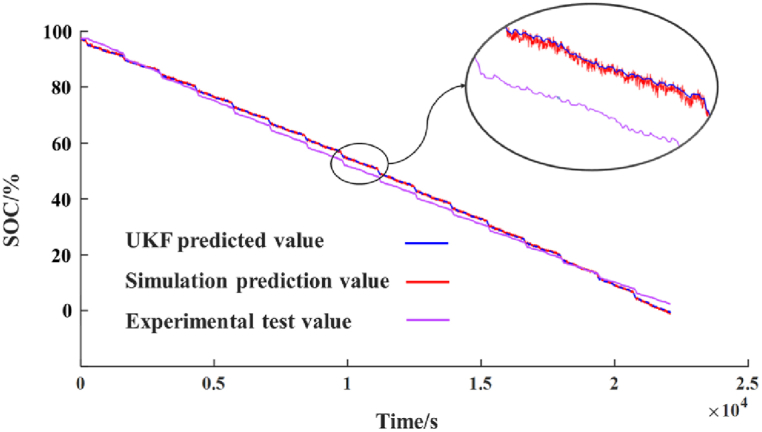
Fig. 8: Comparison of battery SOC predicted by UKF and experimental test results.

**Fig. 9:**
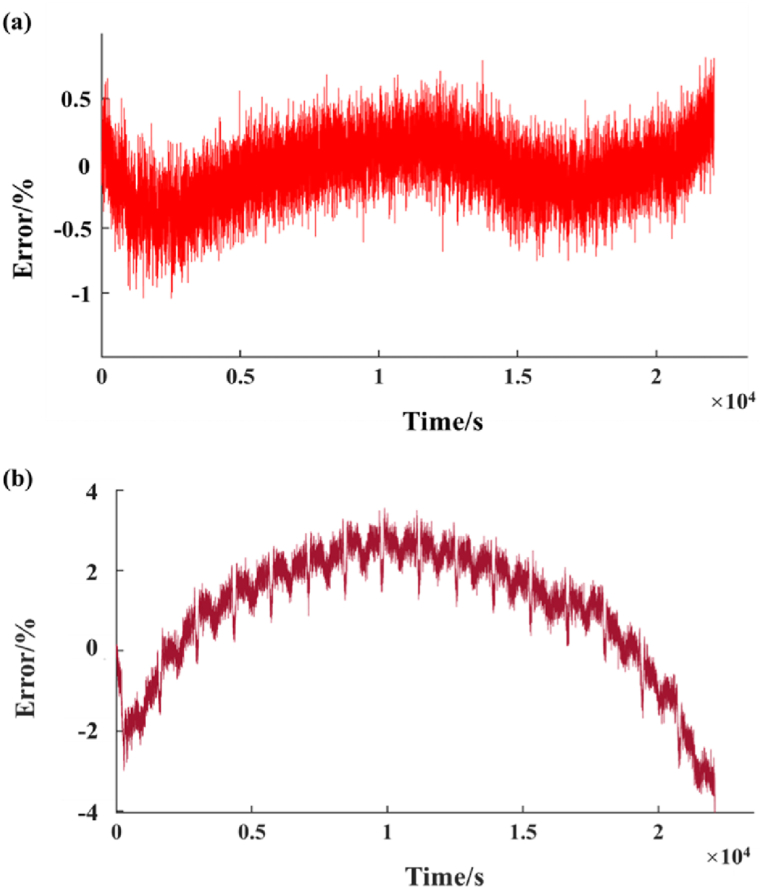
Error curve: (a) The error between UKF prediction results and simulation prediction results; (b) The error between UKF prediction results and experimental test results.

The predicted results of battery SOC obtained by untraced Kalman filtering algorithm are shown in Fig. 8. The error between UKF prediction results and Simulink simulation prediction results are shown in Fig. 9(a), and the error between UKF prediction results and experimental test results is shown in Fig. 9(b).

The prediction accuracy of battery operating state depends on the prediction accuracy of battery SOC. The error between the SOC simulation results and the experimental results of the battery equivalent circuit model established by Simulink fluctuates between −5% and 5 %, but when the battery capacity is about to run out, the error can reach about 20 %.

However, when the untracked Kalman filter is used to predict the SOC of the battery, the error between the SOC results and the experimental results fluctuates between 0.5 % and 0 %, and the maximum error is not more than 1 %. The error between SOC and simulation results fluctuates between ±4 %.

**Fig. 10:**
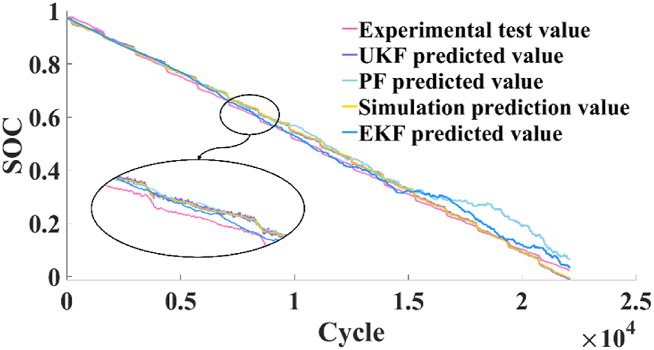
Comparing SOC prediction values using various methods.

To elucidate the rationale for selecting the UKF for adjustment purposes, a comparative analysis was conducted against other pertinent methodologies, with the findings detailed in Table 3, focusing on the rectification of the SOC prognostications for model. Fig. 10 graphically represents the outcomes of this comparison. Moreover, to quantitatively assess the accuracy of these methods, the Root Mean Squared Error (RMSE) associated with each technique are systematically tabulated in Table 4. As mentioned before, since the battery is a non-linear system, the Kalman filtering method is not compared here.

**Table 3:** Comparison table of different methods.

Method	Applicability	Operation principle	Speed of calculate
Kalman Filter	Linear system	Direct solver	Quickly
Extended Kalman Filter	nonlinear systems	Taylor expansion	Moderate
Particle Filter	Linear and nonlinear systems	Monte Carlo integration	Very slowly
Unscented Kalman Filter	Linear and nonlinear systems	Unscented transformation	Moderate

**Table 4:** RMSE of different methods.

Method	Compare with simulation signal	Compare with test signal
Extended Kalman Filter	0.0446	0.0465
Particle Filter	0.0032	0.0214
Unscented Kalman Filter	0.0025	0.0186

The results presented in Table 4 provide a comprehensive comparison of the RMSE values, contrasting the forecasted SOC obtained through UKF correction with both simulated predictions and experimental data. Notably, the UKF demonstrates exceptional precision, yielding RMSE values of 0.0025 and 0.0186 when compared against simulated and experimental signals. These findings not only represent numerical measures but also signify the remarkable capability of UKF to maintain minimal error margins, thus affirming its superior accuracy in SOC tracking. Such metrics are not merely numerical but are a testament to ability of the UKF to sustain error margins within a minimal bracket, thereby underscoring its finesse in tracking the SOC with heightened accuracy.

Moreover, the fidelity of the UKF is further exemplified in Fig. 10, which elucidates tracking trajectory of the filter, marked by negligible perturbations, and an unwavering adherence to the experimental curve. This steadfastness is emblematic of robust error covariance handling for the UKF, allowing it to mitigate the propagation of uncertainties throughout the estimation process.

Conversely, the Extended Kalman Filter (EKF), while demonstrating laudable precision, exhibits a subtly higher RMSE value, which, in the graphical representation, translates to a slightly more pronounced oscillatory behavior around the ground truth values. Such fluctuations, although contained, hint at comparatively less precise convergence of the PF to the SOC path.

Similarly, the PF, despite its capability to chart the overarching SOC trend, portrays the most significant departures from the test values, as evidenced in Fig. 10 by its broader error band. This variability, observable as the most pronounced waveforms around the true SOC trajectory, indicates a higher susceptibility to model and measurement noises, affecting its predictive consistency.

Therefore, the prediction of battery SOC by UKF has higher accuracy, which provides higher precision prediction results for battery management system, and provides safety guarantee for the smooth operation of electric vehicle.

## 5 Conclusion

This study deepens the comprehension of the charge-discharge behaviors exhibited by Lithium Iron Phosphate (LFP) batteries through the introduction of an innovative three-stage methodology rooted in model-data fusion. In conclusion, this study has established a robust three-stage method for the accurate estimation of the SOC in lithium-ion batteries, crucial for the operational stability of electric vehicles. By integrating the FFRLS method for parameter identification, a modal-data fusion approach for simulation model construction, and corrections via the UKF, this methodology demonstrates superior precision and reliability. Comparative analyses underscore efficacy of the UKF, yielding the lowest RMSE values of 0.0025 for simulations and 0.0186 for experimental data, while consistently maintaining errors below 5 % against actual data. These findings pave the way for future advancements in battery management systems.

Future research should address the intricate effects of temperature, operational conditions, and other environmental influences on battery performance. Efforts to extend the adaptability of this methodology to diverse scenarios will not only enrich its robustness and applicability but also pave the way for a more nuanced understanding of battery dynamics. Anticipated developments may include innovative adaptive strategies for energy management, tailored to meet the evolving technological demands and environmental considerations.

## Data availability

Data will be made available on request.

## CRediT authorship contribution statement

**Jinrui Zhang:** Writing – original draft, Visualization, Software, Investigation, Data curation. **Chenqi Song:** Software, Data curation. **Jiawei Xiang:** Writing – review & editing, Supervision, Methodology, Funding acquisition, Conceptualization.

## Declaration of competing interest

The authors declare that they have no known competing financial interests or personal relationships that could have appeared to influence the work reported in this paper.
